# Can Emotional Intelligence Be Fostered? The Perspective of Social Learning Theory

**DOI:** 10.3389/fnbeh.2022.862360

**Published:** 2022-03-15

**Authors:** Show-Jane Yen

**Affiliations:** Department of Computer Science and Information Engineering, Ming Chuan University, Taoyuan City, Taiwan

**Keywords:** emotional intelligence, Emotional Resource Deficient Behavior, job performance, positive organizational behavior, leadership

## Introduction

Many studies are investigating how to improve employee positive behavior (Huang et al., 2021, 2022; Zhu et al., 2021). However, these studies ignore the changing perspective of emotional intelligence and instead employ a trait perspective of emotional intelligence (Agnoli et al., 2019; Alegre et al., 2019; Kun et al., 2019) to examine its impact on positive employee behavior, including well-being, job satisfaction and trust (Knight et al., 2015; Di Fabio and Kenny, 2016; Peláez-Fernández et al., 2021). Emotional intelligence refers to people's ability to use and regulate emotions and to evaluate other people's and self-emotions (Salovey and Mayer, 1990). Furthermore, emotional intelligence is not a trivial concept as it can not only increase positive employee behaviors (Knight et al., 2015; Lopez-Zafra et al., 2019; Gong et al., 2020) but also reduce negative employee behaviors (Domínguez-García and Fernández-Berrocal, 2018; Sarrionandia et al., 2018; Fiorilli et al., 2019), which can effectively improve an organization's competitive advantage. Therefore, this article uses leadership to explore its impact on the development of emotional intelligence. Leadership refers to leaders managing the subordinates by showing consideration, understanding, and respect for their needs and feelings (Bass, 1990). This paper proposes a possible relationship that leaders can increase employees' emotional intelligence as they learn from leaders, opening the black box between these two variables.

Furthermore, the present article identifies emotional intelligence as a key resource for employees, because high levels of emotional intelligence mean that these employees have more emotional resources and emotional capacity to not only deal with negative behaviors (e.g., Emotional Resource Deficient Behavior) but also display positive behaviors (e.g., job performance). Indeed, emotional intelligence is the ability to manage emotions so that these employees can use these emotional resources for their own benefit (Salovey and Mayer, 1990). Therefore, this paper proposes that emotional intelligence should affect job performance and Emotional Resource Deficient Behavior, as more resources should lead to higher job performance (Huang et al., 2022) and lower Emotional Resource Deficient Behavior (Zeng et al., 2020).

Finally, this article argues that emotional intelligence can change because the definition of emotional intelligence has taken on a changing nature (Salovey and Mayer, 1990). Emotional intelligence refers to the ability to manage emotions in a variety of situations, and this ability is one that people can learn from others (Bandura, 1986), supporting that emotional intelligence can change over time. Indeed, Nelis et al. (2009) found that emotional intelligence can be cultivated through education and training, thereby supporting a change in perceptions of emotional intelligence.

## Literature Reviewing

**Figure 1:**
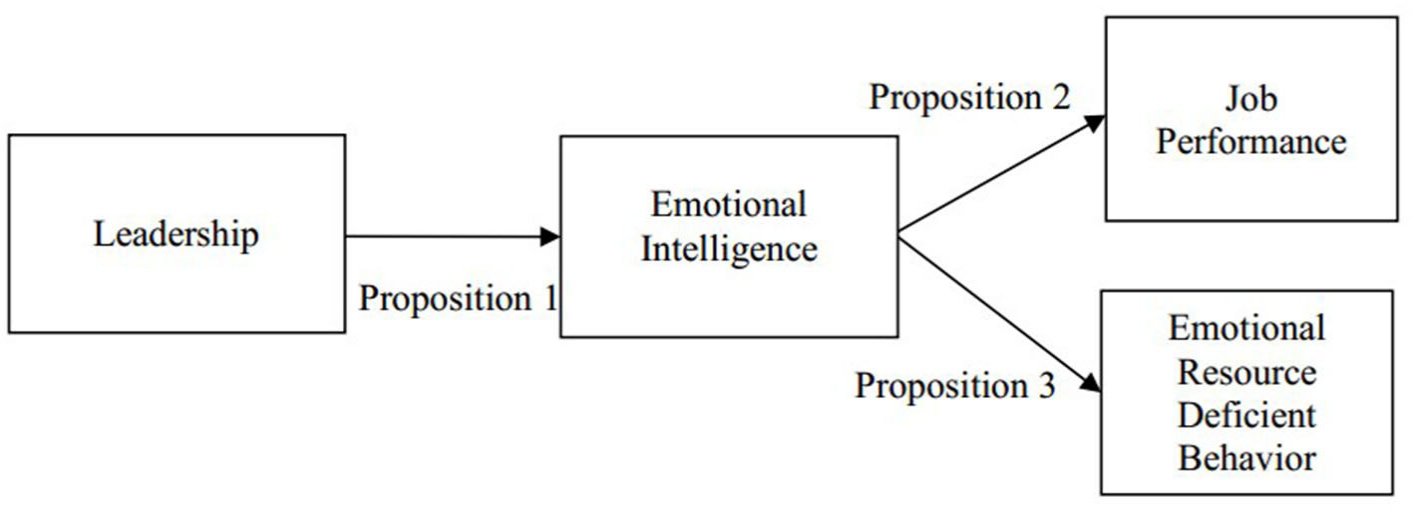
Research framework.

This article proposes a new framework, shown in Figure 1, that leadership influences emotional intelligence development, which in turn influences job performance development and Emotional Resource Deficient Behavior development.

### Emotional Leadership and Emotional Intelligence

Emotional intelligence refers to the ability to use and regulate emotions and to evaluate the emotions of others and one's own emotions (Salovey and Mayer, 1990). Based on social learning theory (Bandura, 1986), subordinates can learn a leader's ability to assess and manage emotions, similar to the field of emotional intelligence. Social learning theory (Bandura, 1986) argues that individuals passively learn the behavior of others in a group because they can be rewarded, and these individuals avoid exhibiting behaviors that would be punished. For example, these subordinates who learn their superiors' ability to manage their emotions will inevitably be positively appreciated by their superiors, thereby supporting the relationship between leadership and emotional intelligence. Indeed, previous research has demonstrated that subordinates can learn behaviors from leaders and then exhibit those behaviors in workgroups (Zhang et al., 2020), and emotional intelligence is a learned ability (Mayer et al., 2008).

Proposition 1: A leader's emotional leadership should affect the emotional intelligence development of subordinates.

### Emotional Intelligence Development, Job Performance Development, and Emotional Resource Deficient Behavior Development

As mentioned above, emotional intelligence is a resource that can improve employee performance and reduce negative employee behavior. Indeed, past articles have demonstrated that personal resources can improve performance, as more emotional resources should give employees more opportunities to achieve higher performance (Bangun et al., 2021). For example, employees have more emotional resources to serve customers, which is bound to attract more customers to buy products. On the other hand, more emotional resources should help employees deal with more negative behaviors (Golonka et al., 2017). For example, employees have more emotional resources to deal with various adverse conditions, which will inevitably reduce the stress and Emotional Resource Deficient Behavior of these employees.

Proposition 2: Emotional intelligence development should cause positive job performance development.

Proposition 3: Emotional intelligence development should cause negative Emotional Resource Deficient Behavior development.

## Discussion

The present article uses social learning theory (Bandura, 1986) to describe why leaders can promote the development of emotional intelligence in their subordinates, which in turn leads to positive job performance development and negative Emotional Resource Deficient Behavior development. Indeed, past research has overlooked the critical role of leadership in shaping followers' emotional intelligence, and this article will advance the literature on leadership and emotional intelligence.

Few studies have explored why leadership can deeply develop emotional intelligence, so the present article creates a new paradigm shift in developing emotional intelligence. This paradigm will guide enterprises on how to improve employees' emotional intelligence through organizational leadership mechanisms to achieve organizational performance.

Finally, to increase competitive advantage, contemporary enterprises should think deeply about how to improve employee performance, and the present article proposes a possible approach, namely emotional intelligence. Indeed, emotional intelligence has been shown to have an impact on both positive and negative employee behaviors and is seen as an important asset in contemporary organizations (Drigas and Papoutsi, 2019). Therefore, contemporary enterprises should use leadership as an important organizational management mechanism because it can promote the development of emotional intelligence.

## Author Contributions

The author confirms being the sole contributor of this work and has approved it for publication.

## Conflict of Interest

The author declares that the research was conducted in the absence of any commercial or financial relationships that could be construed as a potential conflict of interest.

## Publisher's Note

All claims expressed in this article are solely those of the authors and do not necessarily represent those of their affiliated organizations, or those of the publisher, the editors and the reviewers. Any product that may be evaluated in this article, or claim that may be made by its manufacturer, is not guaranteed or endorsed by the publisher.
